# Bayesian-frequentist hybrid inference framework for single cell RNA-seq analyses

**DOI:** 10.21203/rs.3.rs-3384541/v1

**Published:** 2023-10-03

**Authors:** Gang Han, Dongyan Yan, Zhe Sun, Jiyuan Fang, Xinyue chang, Lucas Wilson, Yushi Liu

**Affiliations:** a Epidemiology & Biostatistics, 212 Adriance Lab Rd, 1266 TAMU College Station, TX 77843; b Eli Lilly and Company Corporate Center, 893 Delaware St, Indianapolis, IN 46225

**Keywords:** Bayesian-frequentist hybrid inference, informative prior, single-cell RNA-seq

## Abstract

**Background:**

Single cell RNA sequencing technology (scRNA-seq) has been proven useful in understanding cell-specific disease mechanisms. However, identifying genes of interest remains a key challenge. Pseudo-bulk methods that pool scRNA-seq counts in the same biological replicates have been commonly used to identify differentially expressed genes. However, such methods may lack power due to the limited sample size of scRNA-seq datasets, which can be prohibitively expensive.

**Results:**

Motivated by this, we proposed to use the Bayesian-frequentist hybrid (BFH) framework to increase the power.

**Conclusion:**

In our idiopathic pulmonary fibrosis (IPF) case study, we demonstrated that with a proper informative prior, the BFH approach identified more genes of interest. Furthermore, these genes were reasonable based on the current knowledge of IPF. Thus, the BFH offers a unique and flexible framework for future scRNA-seq analyses.

## 1. Background

Single cell RNA sequencing (scRNA-seq) is a powerful sequencing technology that allows for the profiling of gene expression in individual cells. Traditional bulk RNA sequencing technologies measure the average expression level of all cells in the population, which mask the uniqueness of each cell. In contrast, isolation of cells is an important step in scRNA-seq. It enables the identification of different cell types within complex tissues (Trapnell 2015). As demonstrated in Keren-Shaul et al.’s research, by analyzing immune cell populations in mouse brains, they discovered a novel microglia type associated with neurodegenerative diseases using scRNA-seq (Keren-Shaul et al. 2017).

scRNA-seq has the advantage in processing thousands or even millions of single cells simultaneously (Zheng et al. 2017) and has extensive applications across different fields of biology and medical research. By comparing the gene expression level between patients and healthy controls, scRNA-seq can provide important insights into the disease associated genes and pathways. In drug discovery area, it has become an essential tool to identify novel drug targets and to test the efficacy of drugs on specific cell types. For instance, in the study by Wu et al. (2022) on diabetic kidney disease (DKD), they generated single cell data from nearly 1 million cells and analyzed the response of a murine DKD model to five treatment approaches. They found that different medications affected different cell types and combination therapies achieved better outcomes in rescuing DKD-associated transcriptional changes.

One important question in analyzing scRNA-seq data is the identification of differentially expressed genes (DEG) between groups. Compared to the gene expression data generated from other technologies, scRNA-seq data have some unique features including overdispersion, sparsity, high proportion of zeros due to dropout events (i.e., scRNA-seq data only captures a small fraction of the transcriptome of each cell), and the hierarchical structure embedded in the data (Das et al. 2021). Early scRNA-seq studies often collect many cells from one or a few individuals. With the rapid advancement in the technology, scientists have started to collect single cell data from multiple individuals. In a multi-individual, multi-condition experiment, other than cell-to-cell variation within each individual, heterogeneity also exists among different conditions, individuals and across different cell types. Those distinctive challenges need to be considered when we explore DEG in scRNA-seq data.

With more and more gene expression data becoming publicly available, many approaches and tools for the differential expression (DE) analysis have been developed for scRNA-seq data. For example, ZINB-WaVE (Risso et al. 2018) and ZingeR (Van den Berge et al. 2018) assume the expression counts follow a zero-inflated negative binomial (ZINB) distribution and apply Expectation-Maximization (EM) algorithms to estimate model parameters. In contrast, SCDE (Kharchenko, Silberstein, and Scadden 2014) models the observed abundance using a mixture of the Poisson (dropout component) and negative binomial (amplification component) distribution. These approaches require several distributional assumptions which may fail to be satisfied by real data. Finak et al. (2015) proposed a hurdle model to simultaneously model the expression rate and mean expression values for a specific gene, then DE testing is performed between two cell populations using likelihood ratio test statistic. Non-parametric approaches were also applied on analyzing scRNA-seq data, such as Wilcoxon signed rank test and ROSeq (Gupta et al. 2021), both of which use test statistics based on ranks. In summary, many single-cell-specific DE methods which apply different strategies, have been developed in recent years. However, some existing approaches are inappropriate for individual level differential expression testing (such as comparison between patients and healthy controls), as the sampling units for these approaches are cells, not individuals (Zhang et al. 2022). Failing to account for the intrinsic variability of individuals causes a systematic underestimation of the variance of gene expression, compromising the ability to generate biologically accurate results.

Pseudo-bulk methods, which pool the scRNA-seq counts in the same biological replicate, have been developed to address this variability. Squair et al. (2021) evaluated the performance across fourteen different DE methods using eighteen datasets and found pseudo-bulk methods outperform other cell-level based DE methods in scRNA-seq data. Murphy and Skene (2022) also recommended the use of pseudo-bulk approaches after the simulation analysis from multiple scenarios. Biased inference and highly inflated type 1 error rates were observed when scientists assume cells from the same individual are statistically independent. Zimmerman, Espeland, and Langefeld (2021) proposed a generalized linear mixed model that incorporates a random effect for individual, to address the correlation structure from cells within an individual. Another method IDEAS (Zhang et al. 2022) captures the gene expression profile in each individual by a probability distribution and then compares such distributions across two groups of individuals.

Regardless, pseudo-bulk methods could still lack power to detect genes of interest due to the limited sample size. To overcome these limitations, we propose to use a Bayesian-frequentist hybrid (BFH) inference method to analyze the scRNA-seq data at the individual level. The BFH theoretical framework was originally proposed by Yuan (2009) and the computation framework based on the EM algorithm and Monte-Carlo Markov Chain was proposed by Han et al. (2023). In BFH, part of the model parameters is frequentist, and others are Bayesian. The goal of BFH is to obtain estimation of both types of parameters and quantify the variation in the estimation. BFH is achieved by maximizing the likelihood function given the Bayesian parameters and simultaneously minimizing the posterior expected loss function given the frequentist parameters. We extended the work of Han et al. (2023) using a linear regression model based on normal distribution, where both the frequentist and Bayesian estimators have tractable analytic forms. We also derive the estimation error (or standard error) of the frequentist and Bayesian parameters. With a point estimate and a standard error of an estimator, we can construct confidence intervals of the coefficients, which can also be used to test whether predictors (such as disease group) are significantly associated with gene expression.

## 2. Methods

### 2.1. The hybrid inference in existing literature

BFH inference is designed for models that have both frequentist and Bayesian parameters (Yuan 2009). Suppose the frequentist and Bayesian parameters are $\theta_{A}$ and $\theta_{B}$, respectively, the data is Y, and the prior for $\theta_{B}$ is $\pi \left(\theta_{B}\right)$. Given a decision d(Y), a loss function $W\left(d\left(Y\right),\theta_{B}\right)$, and the distribution likelihood $f\left(Y|\theta_{A},\theta_{B}\right)$, the hybrid estimators of $\theta_{A}$ and $\theta_{B}$ are

$$\left({\hat{\theta }}_{A},{\overset{ˇ}{\theta }}_{B}\right)=\arg infsup\int W\left(d\left(Y\right),\theta_{B}\right)f\left(Y|\theta_{A},\theta_{B}\right)\pi \left(\theta_{B}\right)d\theta_{B},$$

where inf and *sup* were taken in the space of d(Y) and $\theta_{A}$ respectively so that ${\overset{ˇ}{\theta }}_{B}$ minimizes the posterior risk given ${\hat{\theta }}_{A}$ and ${\hat{\theta }}_{A}$ maximizes the likelihood function given ${\overset{ˇ}{\theta }}_{B}$. The frequentist parameter ${\hat{\theta }}_{A}$ is defined (and can be numerically calculated) as integration of the loss function over the posterior distribution. (Yuan 2009) proved that the hybrid estimator is a consistent estimator, and the standard error of the hybrid estimators can converge to that of the frequentist estimators. As a result, the variance-covariance matrix can be quantified using Fisher information matrix. Han et al. (2023) developed an EM computational algorithm to compute $\left({\hat{\theta }}_{A},{\overset{ˇ}{\theta }}_{B}\right)$ for any loss function ensuring that the hybrid inference is applicable to general practical problems and different data settings. Han et al. (2023) demonstrated, in extensive simulation studies, that the hybrid inference based on the EM algorithm can outperform Bayesian inference and frequentist inference. In this paper we adopt the EM algorithm in Han et al. (2023) to make inference. Data, statistical models, and more details about the computation are given in Section 2.4.

### 2.2. Data source

#### Lungmap dataset:

The Lungmap dataset used in this study was from a published human lung tissue study (Wang et al. 2020). The cells were clustered by Seurat v3 (Butler et al. 2018) and annotated to 31 cell types based on canonical lineage-defining markers. Lungmap dataset served as the reference dataset for Hierarchical XGBoost (Chang et al. 2023) algorithm to obtain the probability for a cell being an alveolar macrophage cell.

#### IPF scRNA-seq dataset:

The idiopathic pulmonary fibrosis (IPF) scRNA-seq dataset was obtained from a previous study on pulmonary fibrosis (PF) disease mechanisms and the corresponding cell types in human lung tissues (Habermann et al. 2020). This dataset contains over 114,000 cells from 22 donors who had cell observations. Among these 22 donors, 10 are from the control group and 12 from the PF group. The IPF dataset served as the query data for HierXGB to obtain the probability for a cell being an alveolar macrophage cell.

#### IPF bulk RNA-seq dataset:

We also obtained bulk RNA-seq IPF data from human lung tissues (Furusawa et al. 2020) in the previous research on the relationship between chronic hypersensitivity pneumonitis and idiopathic pulmonary fibrosis. This bulk IPF dataset contains 18,838 genes from 103 idiopathic IPF samples and 103 unaffected controls samples. For each gene, the mean differences of expression level between the IPF and control groups were calculated using linear regression with the adjustment of age, sex, race, and smoking history. This difference served as the informative prior of the phenotype coefficient.

### 2.3. Data preprocessing

For scRNA-seq dataset, we selected genes with average expression level across cells greater than 0.1. We removed cells when the number of detected genes was below the lower 2-percentile or with more than 10% of mitochondrial gene expression. For the cell weight calculation, we aligned Lungmap dataset and IPF single cell RNA-seq datasets by their common genes, resulting in 13,988 common genes and 44,294 and 50,383 cells for Lungmap and IPF, respectively. For coefficient estimation of each gene, we matched the IPF scRNA-seq and IPF bulk datasets and obtained 7,886 common genes.

### 2.4. Frequentist, Bayesian, and hybrid inference in linear regression with conjugate priors

Here, we would like to introduce each of the methods we attempted to identify genes associated with IPF. In linear regression analysis, given the sample size *n* and the number of regression parameters *p*, the data can be arranged in *Y* of dimension *n* × 1 as the response, and *X* of dimension *n* × *p* as the design matrix. The regression parameter in the model *β* can be arranged in a vector of dimension *p* × 1.

In our analysis the outcome *Y* is the weighted average of a gene’s expression for a particular cell type(e.g., TGF-β1 in alveolar macrophage) at the individual level, *p*=1, and *X* the design matrix is composed of a vector of 1s, *X*_1_ the disease group of the individual (control or IPF), and *X*_2_ the predictive probability of each cell belonging to alveolar macrophage averaged per individual with subsequent negative log transformation. The linear model is $Y=X\beta +\varepsilon ,\varepsilon ∼N\left(0,\sigma^{2}I\right)$, where *I* is an identity matrix with dimension n by n. So $Y∼N\left(X\beta ,\sigma^{2}I\right)$. The regression parameters are $\beta =\left(\beta_{0},\beta_{1},\beta_{2}\right)$, which $\beta_{0},\beta_{1}$ and $\beta_{2}$ are the intercept, regression parameter for disease group, and regression parameter for probability of the cell belonging to alveolar macrophage, respectively. The likelihood value given data (Y,X) is

(1) $$P\left(\left(Y,X\right)|\beta \right)=\prod_{i=1}^{n}P\left(y_{i}=N\left(X_{i}\beta ,\sigma^{2}\right)\right).$$

The conjugate prior of the regression parameter can be written as $\pi \left(\beta \right)∼N\left({\text{μ}}_{\beta },\Sigma_{\beta }\right)$. Then the posterior distribution can be derived as

(2) $$P\left(\beta |\left(Y,X\right)\right)\propto \;P\left(\left(Y,X\right)|\beta \right)\pi \left(\beta \right)∼\prod_{i=1}^{n}N\left(X_{i}\beta ,\sigma^{2}I|y_{i}\right)\;\;\;\;\;\;\;\times N\left({\text{μ}}_{\beta },\Sigma_{\beta }\right)∼N\left({\text{μ}}_{\beta }^{new},\Sigma_{\beta }^{new}\right),$$

where ${\text{μ}}_{\beta }^{new}={\left(\Sigma_{\beta }^{-1}+X^{⊤}X\right)}^{-1}\left(\Sigma_{\beta }^{-1}\mu_{\beta }+X^{⊤}Y\right)$ and $\Sigma_{\beta }^{new}={\left(\Sigma_{\beta }^{-1}+X^{⊤}X\right)}^{-1}\sigma^{2}$. Without the prior π(β), the frequentist’s estimate and its variance of β can be written as ${\text{μ}}_{\beta }^{F}={\left(X^{⊤}X\right)}^{-1}\left(X^{⊤}Y\right)$ and $\Sigma_{\beta }^{F}={\left(X^{⊤}X\right)}^{-1}\sigma^{2}$, which is the ordinary least square estimate.

Finally, following the EM algorithm in Han et al. (2023), the hybrid Bayesian analysis can be written in the following iterative procedures:

**[Step 1.]** Initialize parameters $\left(\beta_{0},\beta_{1},\beta_{2}\right)$ from the frequentist estimates as $\left(\beta_{0}^{\left(0\right)},\beta_{1}^{\left(0\right)},\beta_{2}^{\left(0\right)}\right)$, where $\beta_{0}$ is the intercept, and $\left(\beta_{1},\beta_{2}\right)$ are the slope parameters for disease group $X_{1}$ and the predictive probability of each cell belonging to alveolar macrophage averaged per individual with subsequent negative log transformation $X_{2}$, respectively.

**[Step 2.]** Given the current value of frequentist parameters $\left(\beta_{0}^{\left(t\right)},\beta_{2}^{\left(t\right)}\right)$, generate data $y_{i}^{B}=y_{i}-X_{0,i}\beta_{0}^{\left(t\right)}-X_{2,i}\beta_{2}^{\left(t\right)}$. From the regression model $Y^{B}=X_{1}\beta_{1}$, obtain $\beta_{1}^{\left(t+1\right)}$ as the posterior mean of $\beta_{1}$, given a conjugate (normal) prior of $\beta_{1}$.

**[Step 3.]** Given $\beta_{1}^{\left(t+1\right)}$, the posterior mean of $\beta_{1}$, generate data $y_{i}^{F}=y_{i}-X_{1,i}\beta_{1}^{\left(t+1\right)}$. From the regression model $Y^{F}=X_{0}\beta_{0}+X_{2}\beta_{2}$, obtain $\left(\beta_{0}^{\left(t+1\right)},\beta_{2}^{\left(t+1\right)}\right)$ as frequentist estimate of $\left(\beta_{0},\beta_{2}\right)$.

**[Step 4.]** Iterate steps 2-3 as in EM algorithm.

For frequentist, Bayesian, and hybrid inferences can all generate parameter estimates and the corresponding estimation variances. An estimate and its variance are used to construct a 95% confidence interval (estimate minus and plus 1.96 times of the standard error) and to calculate a *p*-value from the two-sided test (by calculating a z-score of estimate divided by the standard error) of whether this value is equal 0 or not based on the underlying normal distribution.

### 2.5. Acquiring weights for cells

Alveolar macrophage cells have been recognized to play a crucial role in the pathogenesis of IPF (Bargagli et al. 2011; Zhang et al. 2018). Rather than analyzing gene expression levels across all cell types, we are specifically interested in the association between gene expression levels and disease group (IPF vs. control) in alveolar macrophage cells. A simple approach to obtain alveolar macrophage gene expression is to take the unweighted average across the annotated alveolar macrophage cells in the original study. However, single-cell data are high-dimensional, and annotations for different cells have varying degrees of uncertainty. To better characterize the alveolar macrophage gene expression levels, we took this uncertainty into account by assigning higher weights to cells that we are more certain of them being alveolar macrophages. We quantified such uncertainty with probabilities of cells being alveolar macrophages calculated by HierXGB method (Chang et al. 2023).

HierXGB is a supervised machine learning algorithm that aims to classify each single cell in the query dataset into one of cell types from a reference dataset. With a pre-defined cell-type hierarchical tree structure, the algorithm annotates the cell from ancestor to one of descendant subtypes iteratively until reaching the bottom layer. For dataset with a clear cell type hierarchy, including Lungmap, it outperforms other state-of-arts methods in terms of both accuracy and efficiency (Chang et al. 2023). In the analysis, we first had Lungmap and IPF scRNA-seq datasets aligned using batch effect correction (Welch et al. 2018). Then the HierXGB model was trained by Lungmap and produced the predictive probability of a cell belonging to alveolar macrophage for the IPF scRNA-seq data. The obtained probabilities were used as the weights when we combined gene expression across cells to obtain cell-type-specific expression summary for each gene per individual.

### 2.6. Generating predictor X_2_ and outcome Y

In our example, we averaged the probability of being in alveolar macrophages across cells within each of the 22 donors and generated a length-22 vector as the predictor ***X***_**2**_. A higher ***X***_**2**_ indicates that the cells from this donor are more likely from alveolar macrophages. We also used this alveolar macrophage probability to generate outcome Y. For each gene, Y was also a length-22 vector, where the value was the weighted average of counts in this gene across cells. The weights were alveolar macrophage probabilities, and such weighted averages are called pseudo-bulk counts in single cell data analysis. A higher value of Y indicates that this gene expresses highly in alveolar macrophage cells.

### 2.7. Acquiring priors of β and Σ

We use non-informative priors such as (0,0,0) and *diag*(100,100,100) for ${\text{μ}}_{\beta }$ and $\Sigma_{\beta }$, respectively, when we have little information on parameters. However, for RNA-sequencing data, bulk RNA-seq data which are characterized by its affordability and wide availability, can serve as good informative priors. Although bulk data may not have the same high-resolution as single-cell data, they still provide the overall expression level of each gene within the targeted tissue. In our analysis, the key parameter is $\beta_{1}$ for the disease group, and we incorporated bulk RNA-seq into its estimation. For each gene, we used the difference in mean expression levels between IPF and control samples as the prior of $\beta_{1}$. We obtained the difference from the coefficient of IPF indicator in a linear regression model adjusting for other covariates including age, smoke, sex, and race. We observed that the sample variance of the difference was relatively small compared with the magnitude of the difference. Directly using it as the prior for the variance of $\beta_{1}$ would result in a very strong prior distribution concentrated around the mean. To offer a prior with more moderate dispersion, we used the squared root of sample variance of difference as the variance for the prior. For intercept and average negative log expression, we had no prior information and hence kept the non-informative priors for $\left(\beta_{0},\beta_{2}\right)$.

### 2.8. Pathway analysis

Pathway analysis is usually performed using a set of selected features, in this case, differentially expressed genes (Khatri, Sirota, and Butte 2012). The goal of the analysis is to identify common biological pathways or networks and analyze how they interact to form biological processes. The analysis typically involves comparing a list of genes of interest to a reference database that contains information about functional categories such as biological pathways, molecular interactions, canonical signaling, disease biomarker and other areas of biomedical knowledge. The analysis determines whether the genes in the list are significantly enriched or depleted in any of the categories, compared with what would be observed by chance. A pathway with significantly enriched genes would yield a significantly small p-value. We further studied pathways based on a gene subset that satisfies the following criteria. For a set of differentially expressed genes detected by each method, cut-off points were set to obtain a gene subset with FDR less than 0.01 and absolute estimated difference greater than 0.585. The reference database we used was the R package metabaser that collects all system biology products including MetaCore, MetaDrug, and others (Nikolsky et al. 2009; Bureeva et al. 2009). The final pathways were identified based on a p-value less than 0.05. Please see the top10 and detailed summary of pathways in Table 5 and 6 and supplement material S5 and S6.

## 3. Results

### 3.1. Analysis of Transforming growth factor beta 1 (TGF-β1) gene

We exemplified and compared the frequentist, Bayesian, and hybrid inferences with and without informative prior. TGF-β pathway is well-known in terms of its role in pulmonary fibrosis, therefore, we used the results of TGF-β1 as an example (Grunwell et al. 2018). In the analysis, the outcome or response variable (y) was the weighted average gene expression level per individual (see Section 2.5 and 2.6 for details). The two independent variables include 1) whether the individual was in the control or IPF group $\left(X_{1}\right)$ and 2) the average negative log probability of being the macrophage cell $\left(X_{2}\right)$. The model parameters $\left(\beta_{0},\beta_{1},\beta_{2}\right)$ were intercept, coefficients for $X_{1}$ and $X_{2}$, in the regression model, respectively.

**Figure 1.**
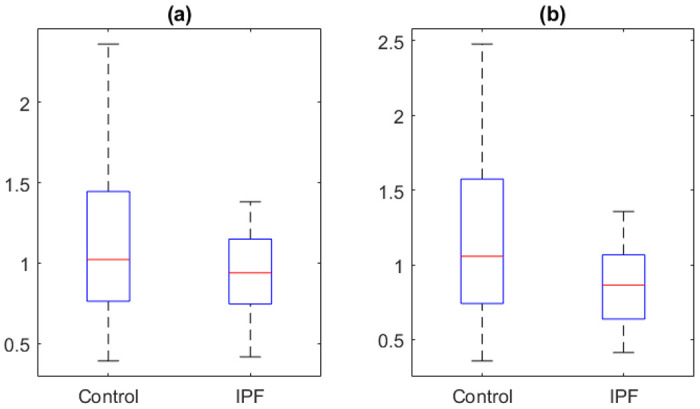
Boxplot of individual level TGF-β1 gene expression by phenotype in the whole sample (a) and predicted alveolar macrophages (b)

Figure 1 shows boxplots of average of gene expression per person grouped by IPF or control. In this sample 12 patients were in the IPF group and 10 were in the control group. Panel (a) has the average gene expressions of all cells for each person, weighted by the probability of each cell being alveolar macrophage cell. Panel (b) has the average gene expression from the cells that were predicted to be alveolar macrophages based on HierXGB prediction. In both (a) and (b), the expression of TGF-β1 in IPF is lower than control, but not statistically significant. The averages of gene expression for control in (a) and (b) are 1.21 and 1.22, respectively. The averages of gene expression for IPF in (a) and (b) are 0.93 and 0.86, respectively. Numerically the expressions from IPF are lower than from control, but Wilcoxon rank sum test p-values are 0.448 for (a) and 0.419 for (b), both are not statistically significant.

Table 1 is a summary of analysis result for gene TGF-β1 and model coefficient for disease group (i.e. IPF and control) $\left(\beta_{1}\right)$, including in the columns the coefficient estimate, standard error, 95% confidence interval, and p-value for testing if the estimated coefficient is different from 0. The linear regression also included intercept and gene probability of being microphage data as a covariate. The five rows in Table 1 correspond to 5 inferences about $\beta_{1}$:

Frequentist: All model parameters were frequentist parameters, and ordinary least square estimates were reported.

Bayesian inference with non-informative prior: A non-informative prior distribution was imposed on all the parameters $\beta_{0},\beta_{1},\beta_{2}$. Mean and standard deviation of the posterior distribution $\left({\text{μ}}_{\beta }^{new},\Sigma_{\beta }^{new}\right)$ were reported.

Hybrid inference with non-informative prior: The same non-informative prior was imposed on $\beta_{1}$, while $\beta_{0},\beta_{2}$ were frequentist parameters.

The Bayesian and hybrid Bayesian inference with informative prior had the Normal distribution with mean −0.31 and variance 0.096 as the prior for $\beta_{1}$, while $\beta_{0},\beta_{2}$ still had the non-informative priors.

The frequentist and Bayesian analyses for the samples with averaged expression across all predicted alveolar macrophages cells by HierXGB had parameters $\beta_{0},\beta_{1}$ only, and the Bayesian inference was based on the same non-informative prior for $\beta_{0},\beta_{1}$ as in Bayesian inference with non-informative prior.

With non-informative prior, the frequentist with Bayesian inferences resulted in similar estimates of $\beta_{1}$. Bayesian inference had slightly wider 95% confidence interval (CI), (−0.692, 0.147), compared with the 95% CI (−0.695, 0.145) from the frequentist inference. The hybrid inference had a similar estimate of $\beta_{1}$ but less standard error (0.144) and shorter 95% CI (−0.557, 0.008) than the Bayesian inference. The hybrid inference with non-informative was marginally significant with p-value 0.057. Given the informative prior, both Bayesian and hybrid inference showed significant effect on $\beta_{1}$, with p-values of 0.017 and 0.005, respectively. The estimates of $\beta_{1}$ were identical (−0.299), but the standard error from hybrid inference (0.106) was less than that from Bayesian inference (0.126), leading to a smaller, more significant p-value from the hybrid inference. This is consistent with the published literature of TGF-β1’s critical role for pulmonary fibrosis (Grunwell et al. 2018).

We also conducted analysis on the samples with average expression of cells predicted as alveolar macrophage by HierXGB. In this analysis the probability of alveolar macrophage prediction was no longer used but the expression was averaged across identified alveolar macrophages cell types. This analysis was consistent with the traditional pseudo bulk analysis, ignoring the predictive probability of cell identity. The frequentist regression analysis p-value is equivalent to that from the two-sample t-test, because the independent variable phenotype is binary, and the F-statistic from regression (or ANOVA) is the square of the t-statistic in the t-test. In this analysis, the Bayesian inference with non-informative prior had similar results as frequentist and both were not significant. Such inference was worse than BFH method with informative prior.

As a result, the analysis of TGF-β1 gene indicates that BFH inference outperforms both frequentist and Bayesian inference. The inclusion of cell type predicted probability for all the cells (regardless how small the probability was), and the informative prior were all valuable for identifying potential significant genes.

### 3.2. Analysis of all genes

We applied each of the five methods to the IPF single cell dataset. The hybrid method with an informative prior detected the largest quantity of genes and biological meaningful pathways. Table 2 summarizes the number of genes detected by each method. See detailed estimation, standard error, and p-value etc. in Table S2-S4. The hybrid method with an informative prior has the highest power with 436 genes detected. Compared with the Bayesian method with an informative prior that discovered 416 genes, the hybrid method detected all of them with an additional 20 genes (Table S1). Among the 20 genes, TREM1 and CCL24 are the most interesting discoveries. Multiple studies have shown their association with IPF. TREM-1 is a receptor expressed on myeloid cells that could serve as an inflammatory biomarker. For example, Dong et al. (2017) studied a highly selective inhibitor to suppress TREM-1 expression and inflammation in murine macrophage. A previous study by Xiong et al. (2022) found that TREM-1 was upregulated in bleomycin (BLM)-induced pulmonary fibrosis (PF) mouse model. They further discovered a pro-fibrotic effect of TREM-1 in PF, a potential strategy for treating fibrotic diseases could be provided. CCL24 protein promotes immune cell trafficking and activation as well as activities that lead to fibrosis. Kohan et al. (2010) revealed that eotaxin-2, the protein encodes by CCL24, stimulated human lung fibroblast proliferation. Mor et al. (2019) concluded that CCL24 plays an important role in skin and lung inflammation and fibrosis pathological progression.

The hybrid method with informative discovered most pathways with a total of 38, whereas the Bayesian method discovered 36 (Table 3). The top pathway from each method involves TGF-β1 in fibrosis development. See Table 5–6. TGF-β is a multifunctional cytokine that belongs to the transforming growth factor superfamily and has multiple isoforms, TGF-β1 is one of them. It has been well established that TGF-β1 plays a role in acute respiratory distress syndrome and pulmonary fibrosis (Grunwell et al. 2018). Past publications have studied the role of TGF-β in alveolar macrophages development. For example, Yu et al. (2017) revealed that TGF-β plays an essential role in controlling the origin, development, and survival of alveolar macrophages. Woo, Jeong, and Chung (2021) reviewed the role of TGF-β in alveolar macrophages development, provided new information and insight into its functions. Grunwell et al. (2018) discovered that targeting the TGF-β1 signaling pathway disruption may be a novel therapeutic approach to improve alveolar macrophage function. In comparison, the hybrid method with non-informative method discovered only two pathways, and their linkage to IPF remains unclear (Table 2). In both gene and pathway discoveries, the hybrid method with an informative prior showed supreme detection power against other methods.

## 4. Conclusion and Discussion

Analysing scRNA-seq data has been a challenging topic, especially given the high cost of running the experiments, which typically results in limited sample sizes. Pseudo-bulk methods, which pool scRNA-seq counts per patient per cell-type, have been commonly used for DE gene detection. However, its performance also relies on the sample size, and hence may lack detection power when sample size is limited. A natural way to overcome this challenge is to borrow information from other studies. Here, we have shown that BFH with informative priors should be considered and has advantages over other approaches. BFH can be viewed as an adaption of Bayesian method and can incorporate prior information with potentially less uncertainty compared with Bayesian methods.

In our BFH analysis, we used pseudo-bulk summarization as the response variable. However, the way to calculate pseudo-bulk is still a topic of discussion in the field. While many researchers have used the annotated cell types directly and summarized the data within a particular annotated cell type, such summarization may potentially lose information since the annotation is based on classifiers with certain thresholds to define the cell types. In our study, we derived the cell-type-specific probability for each cell instead of relying on a classifier to define the cell types. We used this probability as the weight to summarize the data to avoid loss of useful information.

To boost the power of our coefficient estimate, we used bulk RNA-seq data as the prior for each gene. While bulk RNA-seq data are not cell-specific, they offer several advantages as informative priors. First, bulk data have better coverage than scRNA-seq data, thus providing prior information on a more compressive gene list than a typical scRNA-seq experiment. Second, it is relatively inexpensive and readily available. As we have summarized the scRNA-seq data into pseudo-bulk format, the prior derived from bulk RNA-seq data is compatible with our scRNA-seq data. In addition, we transformed the sample variance for β_1_ so that the prior would have better dispersion. Properly setting priors remains as an interesting topic and should be explored further. The BFH method inherits flexibility from the Bayesian framework and can be used iteratively to integrate the current results as new prior information with the new data when appropriate.

Our case example demonstrated the substantial increase in detection power of BFH framework when using informative priors. When non-informative priors were employed, either no differentially expressed genes were identified or only a small number were found. The use of informative priors significantly increased the detection power, as evidenced by the reasonable pathway identified for IPF in terms of the underlying mechanism. The work reinforces the importance for TGF-β pathway and cytokines such as TNF and IL1/IL4, which are well-known for their roles in the IPF mechanism (Redente et al. 2014; Borthwick 2016; Groves et al. 2016). Consequently, our work brings valuable biological insight into the IPF disease for researchers.

A potential limitation of the BFH method is its heavy reliance on informative priors. In situations where relevant bulk RNA-seq or scRNA-seq data are unavailable, alternative data types such as methylation data could be considered as prior information. However, developing such priors needs biological justification and consideration of how to align such data types to pseudo-bulk format of scRNA-seq data. When alternative data types are unavailable, the use of non-informative prior for parameters is inevitable. As discussed in literature (Han, Huang, and Yuan 2018; Han et al. 2023), using Bayesian analysis with non-informative prior can lead to estimation bias and incorrect p-values if the sample size is relatively small.

Despite its limitations, the BFH method is a flexible approach with the capability to incorporate informative prior to enhance detection power. The current framework of the BFH method is implemented using conjugate priors, which reduces the computation time and makes it a suitable method for high throughput analyses in the future.

## Figures and Tables

**Table 1.** The estimation, standard error, 95% confidence interval (95% CI), p-value of $\beta_{1}$ from 7 models: frequentist, Bayesian inference with non-informative and informative priors, hybrid inference with non-informative and informative priors for all cells; and frequentist and Bayesian analysis for the predicted alveolar macrophages.

Sample	Method	Estimate	Standard error	95% CI	P-value
All cells weighted by alveolar macrophages predictive probability	Frequentist	−0.275	0.214	(−0.695, 0.145)	0.199
	Bayesian, non-informative	−0.273	0.215	(−0.692, 0.147)	0.201
	Hybrid, non-informative	−0.275	0.144	(−0.557, 0.008)	0.057
	Bayesian, informative	−0.299	0.126	(−0.545, −0.053)	0.017
	Hybrid, informative	−0.299	0.106	(−0.506, −0.092)	0.005
Predicted alveolar macrophages	Frequentist	−0.357	0.228	(−0.804, 0.090)	0.117
	Bayesian, non-informative	−0.356	0.228	(−0.803, 0.091)	0.119

**Table 2.** Genes detected by the five methods.

Frequentist	Bayesian, non-informative	Hybrid, non-informative	Bayesian, informative	Hybrid, informative
1 gene (CD302)	1 gene (CD302)	43 genes	416 genes	436 genes

* Pvalue adjust: Benjamini and Yekutieli (2001) FDR control

* Threshold: adjusted p-value < 0.01 and absolute value of mean estimation >= 0.585

**Table 3.** Pathways detected by the five methods.

Frequentist	Bayesian, non-informative	Hybrid, non-informative	Bayesian, informative	Hybrid, informative
No pathway	No pathway	2 pathways	36 pathways	38 pathways

* Threshold: qvalue < 0.05

**Table 4.** A detailed list of pathways detected by Hybrid, non-informative method.

Hybrid, non-informative							
Pathways	r	R	n	N	Zscore	pvalue	qvalue
Putative pathways of activation of classical complement system in major depressive disorder	4	37	28	12814	13.81785	1.15E-06	0.00175
Development_Role of proteases in hematopoietic stem cell mobilization	3	37	18	12814	12.95844	1.76E-05	0.013401

* Threshold: qvalue < 0.05; r: intersection of intology term with experiment list; R: size of experiment list; n: size of ontology term; N: size of background list; zscore: z-score of enrichment; pvalue: hypergeometric test enrichment p-value; qvalue: FDR-adjusted pvalue.

**Table 5.** Top 10 pathways detected by Bayesian, informative method, ranked by qvalue.

Bayesian, informative							
Pathways	r	R	n	N	Zscore	pvalue	qvalue
Role of TGF-beta 1 in fibrosis development after myocardial infarction	10	219	38	12814	11.72026	5.39E-10	8.23E-07
IL-1 beta- and Endothelin-1-induced fibroblast/ myofibroblast migration and extracellular matrix production in asthmatic airways	8	219	40	12814	8.93905	3.08E-07	0.000173
Cell adhesion_ECM remodeling	9	219	55	12814	8.403013	3.39E-07	0.000173
TGF-beta-induced fibroblast/ myofibroblast migration and extracellular matrix production in asthmatic airways	9	219	60	12814	7.961536	7.33E-07	0.00028
Th2 cytokine- and TNF-alpha-induced profibrotic response in asthmatic airway fibroblasts/ myofibroblasts	8	219	52	12814	7.623879	2.52E-06	0.000771
Immune response_CCL2 signaling	8	219	54	12814	7.445987	3.39E-06	0.000863
TGF-beta 1-mediated induction of EMT in normal and asthmatic airway epithelium	7	219	44	12814	7.279623	8.66E-06	0.00189
Development_Inhibition of angiogenesis and regulation of endothelial cell function by PEDF	8	219	64	12814	6.677022	1.25E-05	0.002377
Immune response_IL-4-responsive genes in type 2 immunity	8	219	70	12814	6.291138	2.43E-05	0.003892
Role of fibroblasts in the sensitization phase of allergic contact dermatitis	5	219	22	12814	7.61249	2.89E-05	0.003892

* Threshold: qvalue < 0.05; r: intersection of intology term with experiment list; R: size of experiment list; n: size of ontology term; N: size of background list; zscore: z-score of enrichment; pvalue: hypergeometric test enrichment p-value; qvalue: FDR-adjusted pvalue.

**Table 6.** Top 10 pathways detected by Hybrid, informative method, ranked by qvalue.

Hybrid, informative							
Pathways	r	R	n	N	Zscore	pvalue	qvalue
Role of TGF-beta 1 in fibrosis development after myocardial infarction	10	236	38	12814	11.23695	1.12E-09	1.71E-06
IL-1 beta- and Endothelin-1-induced fibroblast/ myofibroblast migration and extracellular matrix production in asthmatic airways	8	236	40	12814	8.554393	5.44E-07	0.000324
Cell adhesion_ECM remodeling	9	236	55	12814	8.026844	6.37E-07	0.000324
TGF-beta-induced fibroblast/ myofibroblast migration and extracellular matrix production in asthmatic airways	9	236	60	12814	7.598001	1.37E-06	0.000522
Development_Inhibition of angiogenesis and regulation of endothelial cell function by PEDF	9	236	64	12814	7.289229	2.39E-06	0.000729
Th2 cytokine- and TNF-alpha-induced profibrotic response in asthmatic airway fibroblasts/ myofibroblasts	8	236	52	12814	7.277826	4.4E-06	0.000978
Immune response_Alternative complement pathway	8	236	53	12814	7.190269	5.1E-06	0.000978
Immune response_IL-4-responsive genes in type 2 immunity	9	236	70	12814	6.872979	5.12E-06	0.000978
Immune response_CCL2 signaling	8	236	54	12814	7.104981	5.89E-06	0.001
TGF-beta 1-mediated induction of EMT in normal and asthmatic airway epithelium	7	236	44	12814	6.951711	1.41E-05	0.002153

* Threshold: qvalue < 0.05; r: intersection of intology term with experiment list; R: size of experiment list; n: size of ontology term; N: size of background list; zscore: z-score of enrichment; pvalue: hypergeometric test enrichment p-value; qvalue: FDR-adjusted pvalue.

## Data Availability

Lungmap dataset could be downloaded from GEO database as GSE161382. IPF scRNA-seq dataset could be downloaded from GEO database as GSE135893. IPF bulk RNA-seq dataset could be downloaded from GEO database as GSE150910.
